# Infant skull fracture risk for low height falls

**DOI:** 10.1007/s00414-018-1918-1

**Published:** 2018-09-07

**Authors:** Marzieh Hajiaghamemar, Ingrid S. Lan, Cindy W. Christian, Brittany Coats, Susan S. Margulies

**Affiliations:** 1 Wallace H. Coulter Department of Biomedical Engineering, Georgia Institute of Technology and Emory University, U.A. Whitaker Building, 313 Ferst Drive, Suite 2116, Atlanta, GA 30332-0535 USA; 2 Department of Bioengineering, Stanford University, Shriram Center, 443 Via Ortega, Rm. 119, Stanford, CA 94305 USA; 3 Department of Pediatrics, Children’s Hospital of Philadelphia, University of Pennsylvania, 34th Street and Civic Center Blvd, Philadelphia, PA 191044399 USA; 4 Department of Mechanical Engineering, University of Utah, 1495 E. 100 Street, 1550 MEK, Salt Lake City, UT 84112 USA

**Keywords:** Pediatric traumatic brain injury, Head impact, Anthropomorphic surrogate, Accidental falls, Finite element modeling, Injury risk curve

## Abstract

Skull fractures are common injuries in young children, typically caused by accidental falls and child abuse. The paucity of detailed biomechanical data from real-world trauma in children has hampered development of biomechanical thresholds for skull fracture in infants. The objectives of this study were to identify biomechanical metrics to predict skull fracture, determine threshold values associated with fracture, and develop skull fracture risk curves for low-height falls in infants. To achieve these objectives, we utilized an integrated approach consisting of case evaluation, anthropomorphic reconstruction, and finite element simulation. Four biomechanical candidates for predicting skull fracture were identified (first principal stress, first principal strain, shear stress, and von Mises stress) and evaluated against well-witnessed falls in infants (0–6 months). Among the predictor candidates, first principal stress and strain correlated best with the occurrence of parietal skull fracture. The principal stress and strain thresholds associated with 50 and 95% probability of parietal skull fracture were 25.229 and 36.015 MPa and 0.0464 and 0.0699, respectively. Risk curves using these predictors determined that infant falls from 0.3 m had a low probability (0–54%) to result in parietal skull fracture, particularly with carpet impact (0–1%). Head-first falls from 0.9 m had a high probability of fracture (86–100%) for concrete impact and a moderate probability (34–81%) for carpet impact. Probabilities of fracture in 0.6 m falls were dependent on impact surface. Occipital impacts from 0.9 m onto the concrete also had the potential (27–90% probability) to generate parietal skull fracture. These data represent a multi-faceted biomechanical assessment of infant skull fracture risk and can assist in the differential diagnosis for head trauma in children.

**Electronic supplementary material:**

The online version of this article (10.1007/s00414-018-1918-1) contains supplementary material, which is available to authorized users.

## Introduction

Falls are the leading causes of nonfatal, unintentional injuries in infants ≤ 1 year of age [1] as well as the leading cause of hospital emergency department visits across all age groups [2]. Historically, an accidental fall is the most common history provided for the mechanism of injury in infants diagnosed with abusive head trauma [3]. Therefore, a detailed understanding of the biomechanics of low-height falls in infants and associated skull fracture risk is critical for informing the differential diagnosis between accidental and abusive head injury etiologies, and for identifying strategies for injury mitigation in household and recreational settings.

Given ethical restrictions for conducting controlled falls in children, pediatric biomechanics of falls are investigated using case reports [4, 5], retrospective clinical studies [6–11], anthropomorphic surrogate drop tests [12–14], cadaver drop tests [15–18], or finite element (FE) model simulations [17–24]. By themselves, each of these types of studies have limitations. For example, retrospective studies are limited by the quality of the data, including the consistency and precision with which fall heights are estimated from incident descriptions. Fall height is rarely estimated by considering the head center of gravity and child’s posture prior to falling [11]. Additionally, because falls may be a false history, methods for excluding cases of abuse should be clearly articulated in all retrospective studies to avoid confounding the data [6]. Cadaveric studies seem the most beneficial, but there is often a lack of specifics regarding the tissue preservation and developmental age of the infant. Weber [15, 16] reported 20 parietal skull fractures (unilateral or bilateral) in 50 infant cadavers dropped from 0.82 m onto five different surfaces. The exact positioning of the infants prior to dropping and detailed descriptions of storage conditions of the cadavers is not provided. More recently, Prange et al. [17] and Loyd [18] performed drop tests of cadaver subjects from 0.15 and 0.3 m onto a metal anvil with five different head impact locations and observed no skull fractures in newborns but noted fractures in children 5–22 months of age.

To improve on the limitations of the individual study types, laboratory-based studies are often paired with FE model simulations to identify fracture thresholds. Roth et al. [19, 20] and Miyazaki et al. [5] developed 6-month-old, 17-day-old, and 23-month-old infant head models, respectively, and validated fracture predictions against a single case of fracture for each age. Cao et al. [21] and Zhou et al. [22] validated their 10-year-old child head models against a single case of a fatal pediatric fall involving a subdural hematoma but no skull fracture. Coats et al. [23] validated the fracture behavior of their 1.5-month-old infant head model with a single simulation of Weber’s infant cadaver drops onto stone and a single real-world fall onto the carpet [24]. Li et al. [25] simulated all 50 of Weber’s cadaver drops and subsequently developed pediatric skull fracture risk curves for 0–9 month-old infants using six different biomechanical parameters. As detailed earlier, lack of specifics regarding the tissue sample harvest and preservation methods may compromise the value of using the cadaver data obtained in Weber’s study for fracture threshold assessment. In addition, the influence of the body kinematics to the head impact response was neglected in many of previous FE studies. Experimental studies using anthropomorphic test devices showed that the body motion and posture during fall and at the moment of head impact affect the force applied to the head [26, 27]. Some FE studies which compared the results of the whole-body FE model to its detached head model also showed that the presence of the whole body affect the kinematics of head impacts [28, 29]. Therefore, using a full-body surrogate to reconstruct falls will provide more realistic head impact force-time histories. In summary, skull fracture thresholds for living infants remain a pressing need for the clinical and biomechanics scientific communities.

**Fig. 1:**
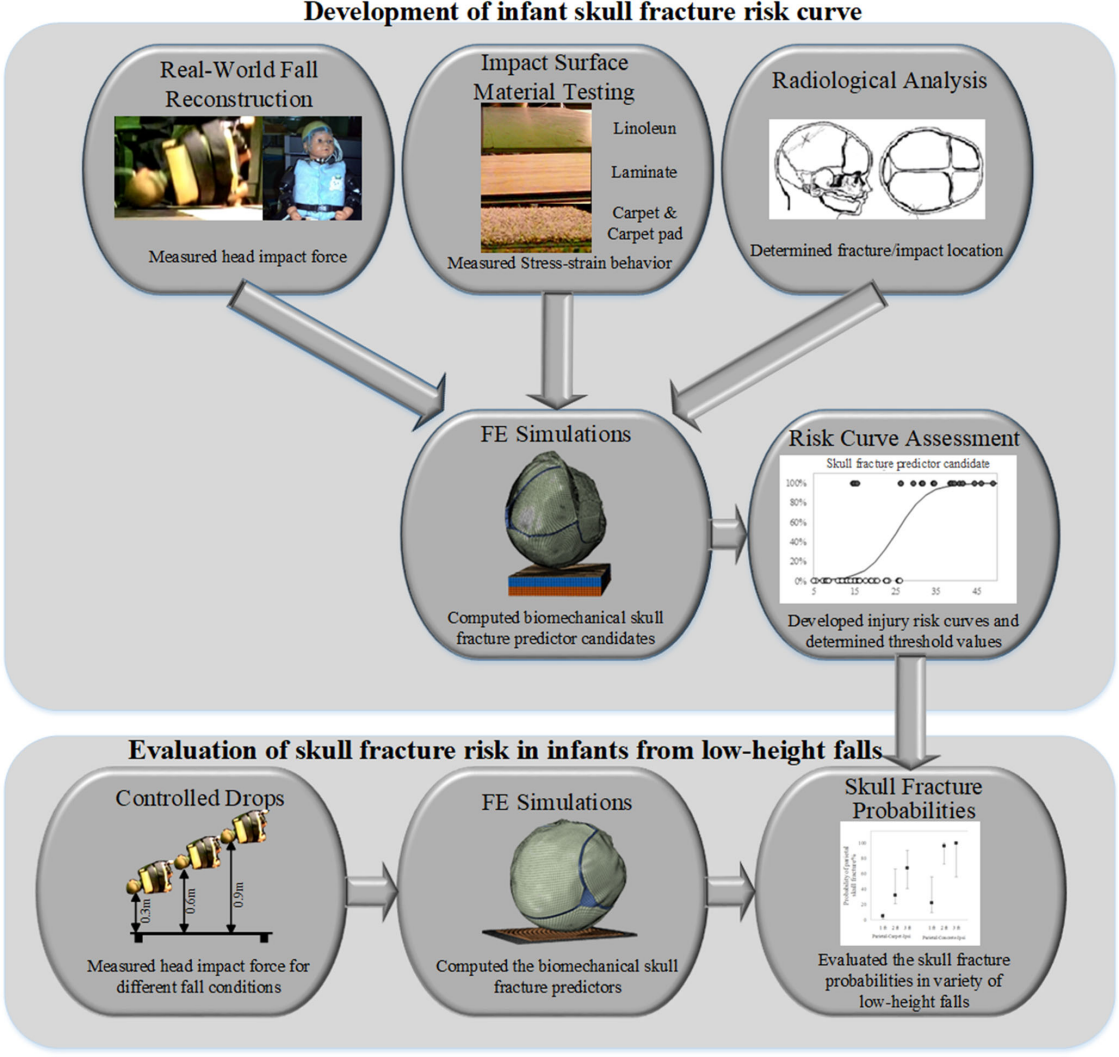
Schematic of the workflow applied in this study to develop infant skull fracture risk curves and evaluate the probability of skull fracture risk from low-height falls in infants

In this study, we utilized an integrated approach combining case-study evaluation, full-body anthropomorphic infant fall reconstruction, and FE simulation to identify the skull fracture risk associated with low-height falls in infants. The infant head FE model incorporates material properties of the human infant skull and suture from children over the age range of 19 days to 4.5 months [30]. Using measured force traces from anthropomorphic reconstructions of 11 well-witnessed infant falls, four FE-based biomechanical parameters were proposed as candidates for predicting skull fracture. Parietal skull fracture risk curves were developed for each candidate using the associated radiological imaging for each case, and further verified with cadaver data [15, 17]. The fracture risk curves were then used to assess the likelihood of parietal skull fracture in various low-height fall scenarios using whole-body anthropomorphic surrogate drop tests and FE simulations. A schematic summarizing the workflow of this study is shown in Fig. 1. Our study is a comprehensive investigation of skull fracture likelihood in short falls in infants, defines skull fracture risk curves for infants and identifies conditions associated with parietal fracture risk following parietal and occipital impacts.

## Materials and methods

### Data collection

To identify biomechanical response metrics and thresholds that are predictive of parietal skull fracture, data from simple well-witnessed falls with detailed event descriptions and radiological reports were examined. The study was approved by the Institutional Review Board of the University of Pennsylvania and Children’s Hospital of Philadelphia (CHOP) and carried out in accordance with the IRB approved guidelines and regulations. All infants less than 6 months of age with a history of a fall (E-codes E880-E888, E987-E988) admitted to the CHOP between June 2006 and September 2009 were evaluated as potential subjects. To be considered for the study, the medical history had to state that the fall event had been witnessed by two adults, one adult and one child old enough to communicate, or one adult whose statement had been designated as without suspicion of abuse by the CHOP child protection team. When the incident was witnessed by two or more persons, the incident description was reviewed carefully to ensure that there was no inconsistency in the details of the incident history. Inclusion criteria also specified that CT and/or MRI scans were obtained within the first week of hospitalization and evaluated by a radiologist. Exclusion criteria were a history of child abuse, mental retardation, drug dependency, birth complications, hemophilia, and pre-existing skull or brain abnormalities (e.g., osteogenesis imperfecta). Cases with a history of a complex fall that would be difficult to reconstruct (e.g., falls involving stairs, strollers, shopping carts, tripping, someone falling on top of the child, or a secondary impact like a coffee table or door), and those with ambiguous event descriptions were also excluded. Cases with fall heights greater than 5 ft were excluded due to limits of the test facility. Of 263 cases screened, 44 cases met the inclusion/exclusion criteria. Cases were further evaluated to determine whether the details of the incidents required for reconstruction were explained clearly in the medical records. If needed, care providers and/or parents were contacted following approved consent procedures to obtain additional event information. The required details were the position of child immediately prior to and after the fall, an estimation of the height of the fall, type of flooring that child landed on, the first part of the child’s body to come into contact with the ground, anything else the child came into contact with during or after the fall, the height of the person involved in the fall, and the age of the witnesses. Additional details about the sustained injuries and health conditions of the child prior to the accident were also gathered. Twenty-six of the 44 cases had unambiguous, well-described fall histories. These 26 patient records and images were reviewed carefully by a radiologist to document; if possible, the exact location of skull fracture or other physical signs of head impact, such as soft-tissue swelling or subgaleal hematoma. Cases were excluded from further consideration if images were of poor quality or if no physical signs of head impact were observed. Cases were excluded if there was a lapse of more than 1 day between accident and hospital visit, thus decreasing the efficacy of using the CT/MRI data to determine exact impact location. Based on the medical record, witness and/or interview report, and radiology report, 11 of the 26 subjects were enrolled in the study: 7 with diagnosed parietal skull fracture (none with occipital fracture) and 4 with no skull fracture. Details of each case, including a brief description of the event, age, sex, estimated fall height, impact surface, area of impact, and skull fracture are provided (Table 1). The fall height represents the distance of the head center of gravity to the impact surface throughout this study.

**Table 1:** Summary details of the 11 witnessed real-world accidental falls in infants of age 4 days to 5.5 months

	Description	Age	Weight (kg)	Sex	Fall height (m)	Impact surface	Impact site	Brain injury/skull fracture type	Fracture status	Witness level
Case 0	One of the parents reached for the baby while the other parent was holding her. The baby arched its back and flipped over the parent’s arm. They were unable to catch the baby	4 months	4.5	Female	1.2	Carpet with carpet pad	Right parietal	Small right frontoparietal EDH overlying right-sided minimally depressed skull fracture	Yes	2 adults
Case 8	Rolled off lap while facing down on lap and flipped over, landed head first on the kitchen floor	5 weeks	4.2	Female	0.6	Linoleum	Right parietal	No associated intracranial hemorrhage; no loss of consciousness; comminuted skull fracture	Yes	1 adult
Case 80	Parent was standing up holding the baby. Fell from parent’s arms while facing the other parent.	4 days	3.6	Male	1.5	Linoleum	Vertex	Left parietal SDH and right posterior parietal SDH; SAH in bilateral parietal and temporal regions; no loss of consciousness; long linear fracture coursing obliquely in the right parietal skull and ending in the sagittal suture	Yes	2 adults happened in hospital
Case 98	Fell from the car seat on the kitchen counter (not strapped in) to the tile floor	5.5 months	6.9	Female	0.9	Tile	Left side of the occiput	Small boggy posterior SDH	No	1 adult
Case 94	5-year-old brother picked baby up and accidentally tripped and dropped him on his occiput/backside onto the carpeted floor	2 months	6.84	Male	0.66	Carpet with carpet pad	Occiput	No hemorrhage or swelling	No	2 adults and 1 child
Case 108	Fell out of parent’s left arm while the parent was reaching to get something with the right arm. The baby landed on the back side of the floor	11 weeks	5.6	Female	1.2	Carpet with carpet pad	Occiput	Hyper density along the right frontal cortex; acute SAH; no swelling; no retinal hemorrhages; no midline shift	No	1 adult more adults were in the house
Case 132	Rolled off examining table (32–35″ height) and fell to the office floor	2.5 months	6.0	Female	0.86	Linoleum	Right parietal	No acute intracranial hemorrhage or midline shift; large overlying scalp hematoma; long, nondisplaced right parietal skull fracture	Yes	2 adults
Case 137	Being held by 5-year-old sister on bar stool, she dropped baby onto the linoleum floor onto the right side of the head	3 months	6.2	Male	1.07	Linoleum	Right parietal	No loss of consciousness or intracranial traumatic lesions; overlying scalp soft tissue contusion; nondisplaced right parietal bone fracture	Yes	1 adult and 1 child
Case 183	Fell sideways out from the car seat (not strapped in) onto the cement and landed on the left side	3 weeks	4.7	Male	0.6	Cement	Left parietal/temporal	Left temporal lobe SDH; no midline shifts	No	1 adult and 1 child
Case 238	2-year-old brother dropped the baby onto the hardwood floor	45 days	4.9	Male	0.46	Laminate hardwood	Left parietal	Small SDH at the left frontal lobe, large left parietal scalp hematoma; complex left parietal skull fracture	Yes	1 adult and 1 child
Case 240	Fell from parent’s lap onto the carpet	34 days	3.7	Male	0.46	Carpet with carpet pad	Left side of the sagittal suture	Small SAH in left operculum and left frontal convexity; nondisplaced posterior left frontal skull fracture	Yes	More than 2 adults

*EDH* epidural hemorrhage, *SDH* subdural hemorrhage, *SAH* subarachnoid hemorrhage

### Surrogate reconstruction of real-world accidental falls

To estimate the head impact force of each child, the 11 cases were reconstructed using a whole-body biofidelic anthropomorphic infant surrogate [13]. A six degree of freedom force plate (Model FP4060-07, Bertec, Columbus, OH, USA) was used to measure the head impact force-time history. Whole-body surrogate reconstruction tests are useful to take into consideration the effect of body motion and posture during fall and at the moment of head impact on the force applied to the head. The head of the surrogate was constructed of five copolymer polypropylene plates (*E* = 535 ± 139 MPa) attached together with a silicone material (*E* = 2.1 ± 0.2 MPa) and covered with a 1-mm-thick latex cap that duplicate the mechanical response of the infant skull, suture, and scalp reported in the literature [30]. The surrogate’s total head and body mass was 4.4 kg with the head mass of 1 kg, which approximately represented a 28th percentile male or 47th percentile female 1.5-month-old infant according to CDC Growth Charts [31]. For each of the 11 cases, the head mass of the child was estimated using head to whole-body mass ratio of 0.23 [32], and the head mass of the infant surrogate was matched to the estimated weight by filling the space inside the skull case with a silicone gel with shear properties (*G* = 765 ± 44 Pa) similar to infant brain. There was no air space in the surrogate head. The mass of the body was also adjusted so the overall surrogate mass matched the overall child’s body mass. Additional details on the biofidelity and validation of the surrogate are reported in [13, 14].

The contact surfaces for the 11 cases were concrete, tile, linoleum, laminate, or carpet with carpet pad. The rigid metal surface of the heavy force plate was used as the closest approximation to concrete and tile. Examples of the other three impact surface materials as stated in the history report were obtained and clamped to the force plate. For each case, the primary impact location was determined from the fall description, medical history, and the radiological reports of fracture and soft-tissue swelling. For fall reconstructions, the fall height, flooring, identified head impact location, and the fall description from the medical and witness reports for each case were matched and verified via a high-speed digital video (210 fps, Exilim EX-FC100, Casio). Carbon paper was used to mark impact location on the surrogate head. The nature of accident reconstruction entails some uncertainties, and there is trial-to-trial variability, so it is impossible to determine with absolute certainty the forces applied to the head during the impacts. Creating a corridor of possible responses by performing multiple trials is a way to take these uncertainties into consideration in real-world accident reconstruction studies [33, 34]. Therefore, in order to estimate the range of possible head impact forces experienced during each accident, five fall reconstructions were performed for each of the 11 cases, and has been also used in other real-world accident reconstruction studies. High-speed digital videos of the drops were carefully reviewed, and only the drops that matched the fall details, including the head impact location and orientation as described in witness and radiology reports, were considered acceptable reconstruction drops. The first, second, and third quartile impact force-time histories measured via the force plate from the five acceptable trials were used as the loading conditions for FE simulations of the case. The first, second, and third peak head impact force quartiles (Q1-Q2-Q3) were selected because they are insensitive to outliers.

### Computational reconstruction of real-world accidental falls

Each case was computationally simulated to estimate the stress and strain responses in the skull. A FE head model of a 1.5-month-old infant, developed previously by our group [23], was used as the base for the FE simulations of these accidental falls and scaled according to the head mass of each subject using uniform, isometric scaling of the brain and skull (with scale factor ${\lambda}_x={\lambda}_y={\lambda}_z={\left(\frac{m_{\mathrm{scaled}}}{m_{\mathrm{base}}}\right)}^{1/3}$) [35]. The infant FE head model is composed of sutures (2485 M3D4R membrane elements), brain (11,066 C3D4 solid elements), scalp (1600 C3D8R solid elements), and five skull plates (18,704 SC8R continuum shell elements) [23]. Because the cases involved children from 4 days to 5.5 months of age, we extracted Young’s moduli values for a similar age range from cadaveric specimens tested in three-point bending and published by Coats and Margulies [30]. Specifically, we used 407.27 and 533.43 MPa for the occipital and parietal bones, respectively, which represent averages over the range of 19 days to 4.5 months of age (Table 2). The brain was modeled as a homogeneous isotropic hyperelastic material using the one-term Ogden model and a viscoelastic material using the two-term Prony series as derived in [23]. The brain material parameters used in the FE model included the shear modulus, *μ*_0_; strain-sensitive nonlinear characteristic parameter, *α*; Poisson’s ratio, *υ*; the relaxation moduli, *C*_*1*_ and *C*_*2*_; and time constants, *τ*_*1*_ and *τ*_*2*_, in the Prony series (Table 2).

**Table 2:** Material properties of the infant FE head model components [23, 30]

Parietal bone	Occipital bone	Suture	Scalp	Brain
*ρ* = 2.085 g/cm^3^	*ρ* = 2.085 g/cm^3^	*ρ* = 1.130 g/cm^3^	*ρ* = 1.2 g/cm^3^	*ρ* = 1.04 g/cm^3^
*ν* = 0.19	*ν* = 0.19	*ν* = 0.49	*ν* = 0.42	Ogden model coefficients *μ*_0_ = 559 Pa; *α* = 0.01 Prony series coefficients: C_1_ = 0.3322; C_2_ = 0.3890 *τ*_1_ = 2.9572; *τ*_2_ = 0.1813 *ν* = 0.499
*E* = 533 MPa	*E* = 407 MPa	*E* = 8.1 MPa	*E* = 16.7 MPa	

To model the impact surfaces in the simulations, the linoleum, laminate, carpet, and carpet pad were indented with a 0.076-m platen at 0.02 × 10^−3^ m/s to 2224 N. Depending on available surface area, 5–24 locations on each surface were tested. Tests near boundaries of the flooring sample were excluded. An average stress-strain curve for each material was implemented in the material evaluator of ABAQUS (Version 6.11, Dassault Systèmes Simulia, Providence, RI). Linoleum and laminate were represented with second-order-reduced polynomial models. Carpet and carpet pad were modeled, respectively, with second-order and first-order forms of ABAQUS’s hyperfoam material model. The concrete and tile impact surfaces were represented as rigid bodies. Table 3 provides the material model, density (*ρ*) and coefficients for each flooring surface.

**Table 3:** Material properties of the impact surfaces

Carpet	Carpet pad	Linoleum	Laminate
*ρ* = 0.2 g/cm^3^	*ρ* = 0.09 g/cm^3^	*ρ* = 0.93 g/cm^3^	*ρ* = 0.54 g/cm^3^
Hyperfoam (*N* = 2)	Hyperfoam (*N* = 1)	Reduced polynomial	Reduced polynomial (*N* = 2)
*μ*_1_ = 0.02 kPa	*μ*_1_ = 6.42 kPa	(*N* = 2)	*C*_1_ = 1325 kPa
*α*_1_ = 25.00	*α*_1_ = 8.99	*C*_1_ = 100 kPa	*C*_2_ = 375,234 kPa
*ν*_1_ = 0.30	*ν*_1_ = 0.30	*C*_2_ = 5,000,000 kPa	
*μ*_2_ = 4.64 kPa			
*α*_2_ = 7.38			
*ν*_2_ = 0.30			

**Fig. 2:**
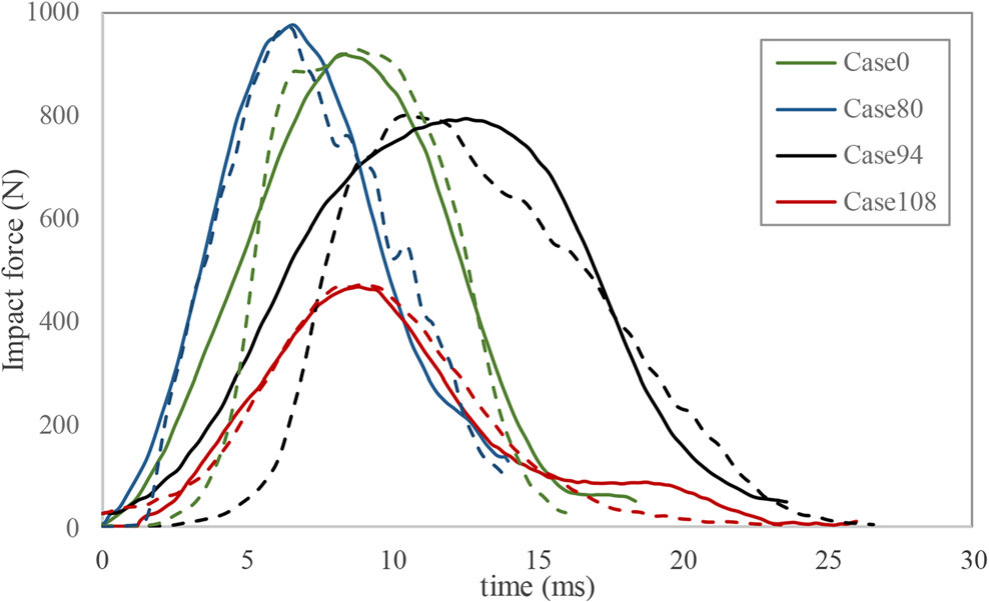
Examples of impact force-time histories extracted from reconstruction experiments (solid) and FE simulations (dashed) for four cases. A comparison between all cases is provided in Table 4

**Fig. 3:**
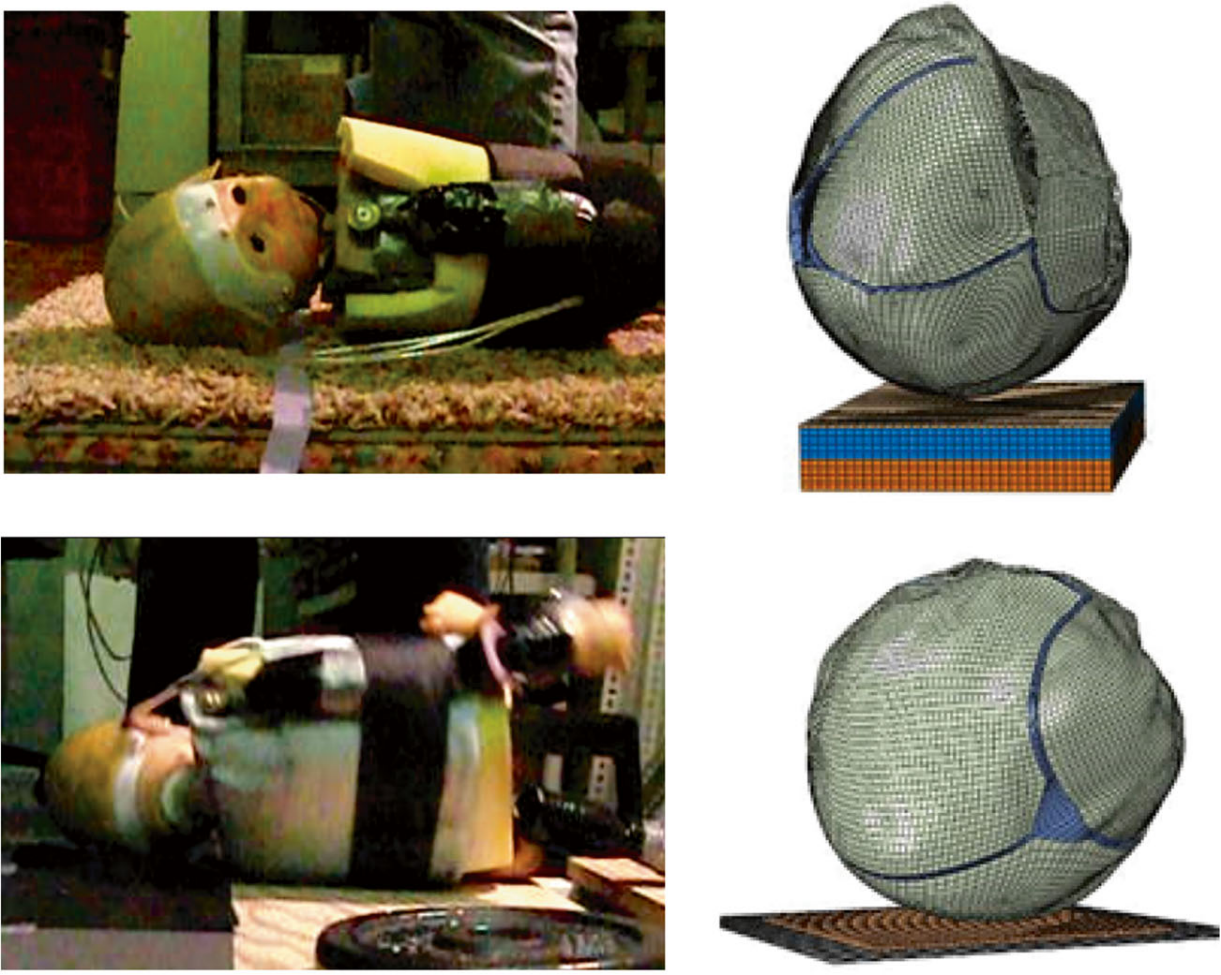
Examples of matching impact location, orientation and surface in FE simulations with reconstruction experiments. The top image is an accidental fall onto carpet with underlying carpet pad and the bottom image is an accidental fall onto concrete

The objective of the FE simulations of the 11 cases was to reproduce both the impact force-time history (Fig. 2) captured in the fall reconstructions as well as the head impact location and orientation as described in witness and radiology reports (Fig. 3). To consider the possible range of head impact forces that might have been applied during each fall, 3 trials (reproducing the first (Q1), second (Q2), and third (Q3) quartile of the measured force-time histories in the reconstruction drops) of each of the 11 cases were simulated (*N* = 33) in ABAQUS explicit. For each FE simulation, the initial velocity of the head was adjusted until the simulated impact force-time history matched the peak and duration of experimental impact force trace within 5% error tolerance (Fig. 2, Table 4). For the seven cases with skull fracture, the distribution of stress and strain in the FE model was compared to the skull fracture location to confirm that high values occurred in the elements located around the site marked as fracture by the neuroradiologist. For some cases, the head orientation and impact location were adjusted slightly, still consistent with the witness reports and videos of reconstruction drops, and the simulation was re-run. As bilateral parietal fractures have been reported in both clinical [36] and cadaveric studies [15, 16], the results of both right and left parietal plates were used in this study. For each simulation, peak temporal values of four fracture predictors (first principal stress, first principal strain, maximum shear stress, and von Mises stress) were extracted from every element in the right and left parietal skull plates.

**Table 4:** Peak impact force comparison (Q1, Q2, Q3) between reconstruction experiments and FE simulations (in parenthesis) for all the 11 accidental fall cases. The peak impact forces from the FE simulations are within 5% error tolerance of the experimental values

Peak impact force (*N*)	Case 0	Case 8	Case 80	Case 94	Case 98	Case 108	Case 132	Case 137	Case183	Case 238	Case 240
Q1	870 (868)	835 (854)	958 (1006)	795 (805)	991 (973)	334 (340)	979 (1010)	798 (785)	378 (383)	453 (443)	329 (332)
Q2	922 (941)	904 (942)	981 (1011)	812 (820)	1001 (1004)	467 (477)	998 (1016)	801 (799)	436 (440)	458 (481)	334 (339)
Q3	1076 (1060)	985 (999)	1005 (1041)	867 (845)	1076 (1022)	554 (535)	1045 (1059)	803 (819)	442 (461)	539 (537)	367 (367)

### Development of skull fracture risk curves for parietal fracture

Each of the 33 simulations generated two peak values for the parietal skull plate (one value from the right plate and another from the left plate) for all 4 candidate predictors. For each of the 4 candidate skull fracture parameters, the 66 generated data points were categorized into one of the three simulation groups representing the first, second, and third peak head impact force quartiles (Q1-Q2-Q3) measured in the reconstruction experiments. For each simulation group, a binary classifier was assigned to each response data point to designate the presence (1) or absence (0) of fracture, based on the radiological report. Binary logistic regression analyses were then performed, and Q1, Q2, and Q3 parietal skull fracture risk curves were created (SPSS v22, IBM, New York, NY, USA) with distribution functions of the following form:

1 $$ P(x)=\frac{e^{a+\mathrm{bx}}}{1+{e}^{a+\mathrm{bx}}} $$

where *P (x)* is the probability of parietal skull fracture for the given value *x* of the predictor candidate. Variables *a* and *b* are the regression coefficients. The quality of fit for the risk curves was then evaluated for the four candidate parameters using the Nagelkerke *R*^2^ and Cox and Snell *R*^2^ statistics to determine the parameter with the highest correlation with skull fracture. Receiver operating characteristic (ROC) curves were also generated, and the area under the ROC curves (AUROC) was used as another measure of how well each parameter distinguished between parietal bones with and without skull fracture. For each candidate fracture parameter, the threshold values corresponding to the 50 and 95% likelihood of skull fracture were extracted from the logistic regression, and the optimal ROC threshold (optimizing specificity and sensitivity) extracted from the ROC curve. For each candidate, the Q2 (or median) risk curve and associated skull fracture threshold value were used for further analysis in this study. The Q1 and Q3 risk curves, and associated threshold values, were used to demonstrate the range of potential uncertainties or errors in injury risk curve development using our real-world accident reconstruction approach.

### Evaluation of skull fracture risk in low-height falls

Using the most robust fracture predictor and threshold determined from the 11 human subject cases, we created an assessment tool for evaluation of the probability of skull fracture in a variety of common low-height household falls. In a previous study [11], we used the same infant surrogate to measure load corridors for head-first falls from three heights (0.3, 0.6, and 0.9 m) onto the concrete or carpet with the underlying carpet pad. Initial head impact was to either the occiput or parietal bone resulting in 120 controlled drops (*N* = 10 drops/condition). The trials producing the minimum, median, and maximum head impact forces for each height-surface-location combination were selected for FE analysis in this study (*N* = 36 simulations, Table 5) using the same infant FE head model described earlier. For each simulation, the initial head velocity was adjusted to match the simulation output force to measured force-time history from corresponding reconstruction drop. The predictors of fracture were extracted from the right and left parietal infant skull plates for each fall condition (height, impact location, and contact surface), and the Q2 skull fracture risk curves for best predictors (maximal principal stress and max principal strain) were used to estimate the likelihood of parietal skull fracture for each fall condition.

**Table 5:** Median and range (minimum and maximum in parenthesis) peak impact force for controlled occipital and parietal falls from 0.3, 0.6, and 0.9 m onto carpet and concrete. The mean and standard error for each combination of head impact location, fall height, and impact surface are published in [13]

Fall conditions	Peak impact force (*N*)		
	0.3 m	0.6 m	0.9 m
Occipital carpet	253 (208–309)	386 (333–413)	494 (443–652)
Occipital concrete	285 (237–360)	488 (404–563)	634.5 (506–736)
Parietal carpet	281 (216–306)	415 (378–511)	531 (450–648)
Parietal concrete	313 (270–387)	509 (423–711)	599 (465–762)

## Results

### Infant skull fracture criteria

**Fig. 4:**
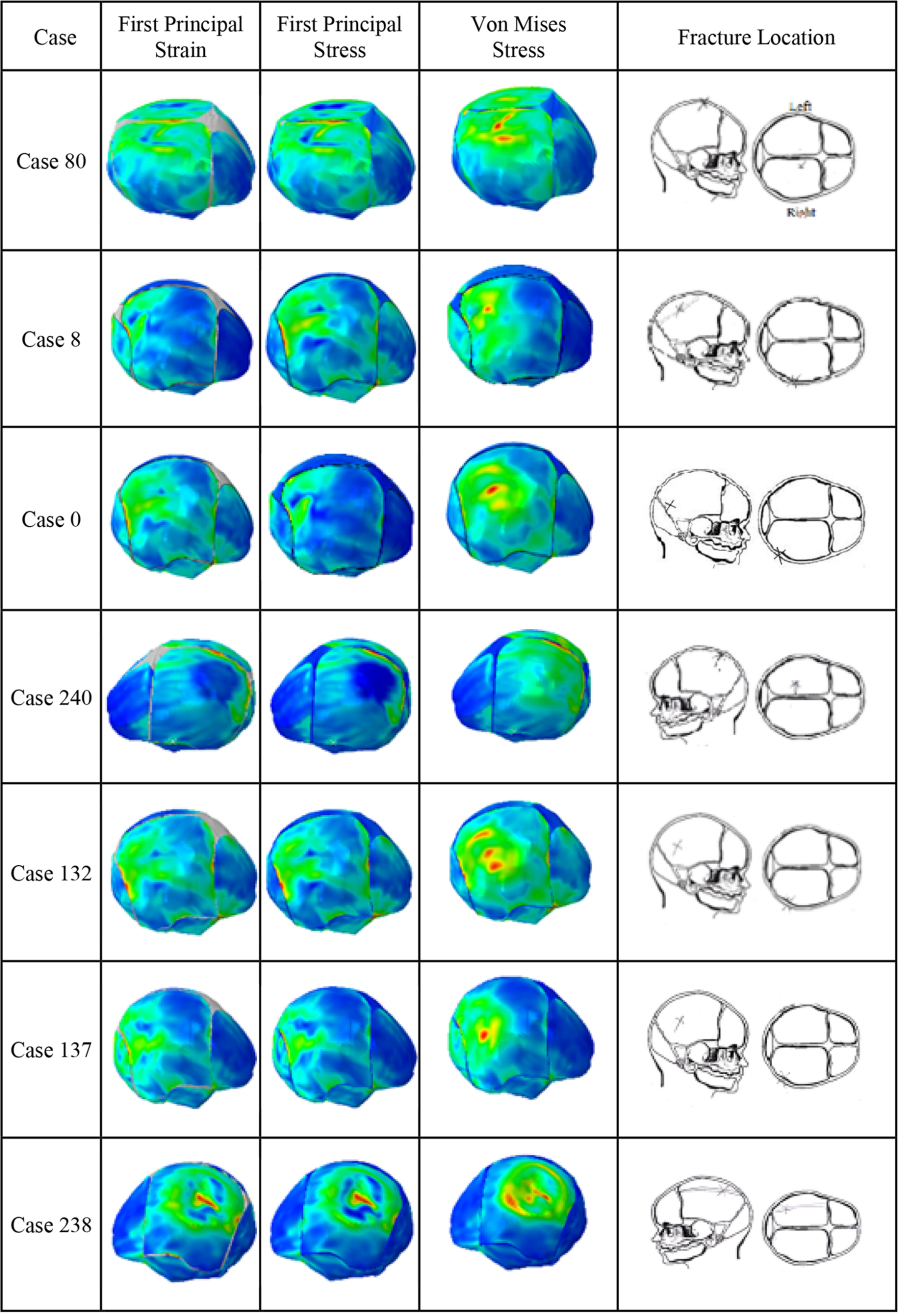
Comparison of first principal stress and strain, and von Mises stress to the skull fracture location for the seven subjects with skull fracture

The distribution of the first principal stress and strain, and von Mises stress, along with the clinical location of fracture, for the seven cases with fracture are shown in Fig. 4. The first, second, and third peak head impact force quartiles (Q1-Q2-Q3) measured in the reconstruction experiments and obtained from the corresponding simulations for all 11 cases are summarized in Table 4. The peak impact forces from the FE simulations are all within 5% error tolerance of the experimental values, indicating that the reconstructed FE simulations are well matched to the surrogate reconstructions.

**Fig. 5:**
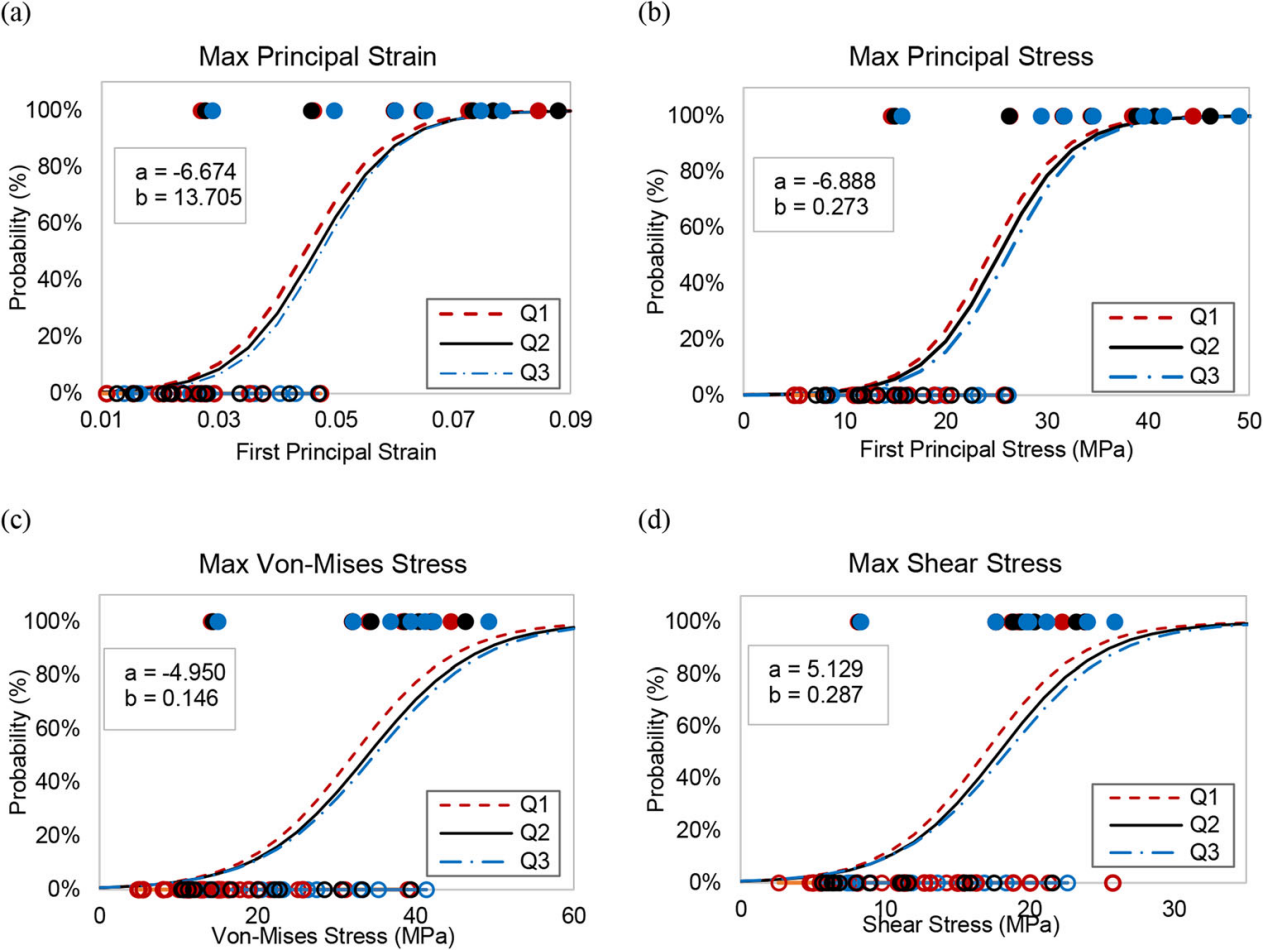
Injury risk curves with associated regression coefficients (**a** and **b** in legend) for infant skull fracture based on maximal first principal stress (**a**), maximal first principal strain (**b**), maximal shear stress (**c**), and maximal von Mises stress (**d**). Presence and absence of parietal fracture in the parietal skull plate are indicated with filled and open symbols respectively

For each potential skull fracture predictor, 66 data points were extracted from the FE simulations (3 simulations per case, 11 cases, 1 maximum data point per simulation from each of 2 parietal skull plates). Each data point was assigned a designation of presence of parietal fracture or absence and is displayed in Fig. S1. The parietal skull fracture risk curves derived from binary logistic regression analysis (Fig. 5) for the four predictor candidates show the least overlap in values for fracture and no fracture for maximal principal stress and strain. Quantitative analysis of the risk curve prediction accuracy for each of the four predictor candidates, evaluated by the AUROC, prediction accuracy rate, the Nagelkerke R^2^, and the Cox and Snell *R*^2^ statistics (Table 6), confirm that the best predictors were first principal stress and first principal strain. These predictors had the highest statistical correlations with AUROC (0.933), prediction accuracy rate (90.9 and 86.4%), Nagelkerke *R*^2^ (0.727 and 0.723), and Cox and Snell *R*^2^ (0.519 and 0.516). The threshold values corresponding to the 50 and 95% probabilities of parietal skull fracture, as determined from the developed injury risk curves for the four predictors are provided in Table 7. Injury threshold values from the ROC analysis demonstrate close agreement with the 50% fracture risk threshold values determined from binary logistic regression curves (Table 7). For each of the four skull fracture predictor candidates, Q1, Q2, and Q3 curves (Fig. 5) demonstrated very similar trends and statistical results (Table 6). Threshold values (Table 7) obtained from these curves are also consistent.

**Table 6:** Statistical results from logistic regression risk curves (columns 2 to 6) and ROC curves (last column) for all potential skull fracture predictors. Data in the first row are from the Q2 curves. Data from the Q1 and Q3 curves are in parenthesis

Potential predictors	Nagelkerke *R*^2^	Cox and Snell *R*^2^	Prediction accuracy rate (%)	Risk curve coefficient *a*	Risk curve coefficient *b*	AUROC
First principal stress (MPa)	0.727 (0.750, 0.744)	0.519 (0.535, 0.529)	90.9 (90.9, 95.5)	**−** 6.888 (− 6.714, − 7.186)	0.273 (0.276, 0.275)	0.933 (0.933, 0.962)
First principal strain	0.723 (0.747, 0.739)	0.516 (0.533, 0.528)	86.4 (90.9, 95.5)	**−** 6.674 (− 6.489, − 6.948)	143.71 (145.18, 149.62)	0.933 (0.933, 0.952)
Shear stress (MPa)	0.460 (0.517, 0.463)	0.329 (0.369, 0.331)	86.4 (90.9, 86.4)	− 5.129 (− 5.108, − 4.966)	0.287 (0.301, 0.269)	0.876 (0.886,0.867)
Von Mises stress (MPa)	0.450 (0.511, 0.453)	0.321 (0.365, 0.324)	86.4 (86.4, 81.8)	− 4.950 (− 4.944, − 4.912)	0.146 (0.154, 0.141)	0.876 (0.876, 0.857)

**Table 7:** Infant skull fracture thresholds based on Q2 injury risk curves and Q2 ROC curves. The range of thresholds based on Q1 and Q3 curves are provided in parenthesis

Potential predictors	50%	95%	ROC
First principal stress (MPa)	25.229 (24.308–26.150)	36.015 (34.968–36.865)	26.083 (26.045–27.789)
First principal strain	0.0464 (0.0447–0.0475)	0.0669 (0.0650–0.672)	0.0439 (0.0418–0.0472)
Shear stress (MPa)	17.898 (16.947–18.571)	28.172 (26.716–29.583)	17.613 (16.859–17.667)
Von Mises stress (MPa)	33.893 (32.027–34.740)	54.056 (51.101–55.562)	31.343 (29.789–31.523)

### Probability of skull fracture in low-height falls

**Fig. 6:**
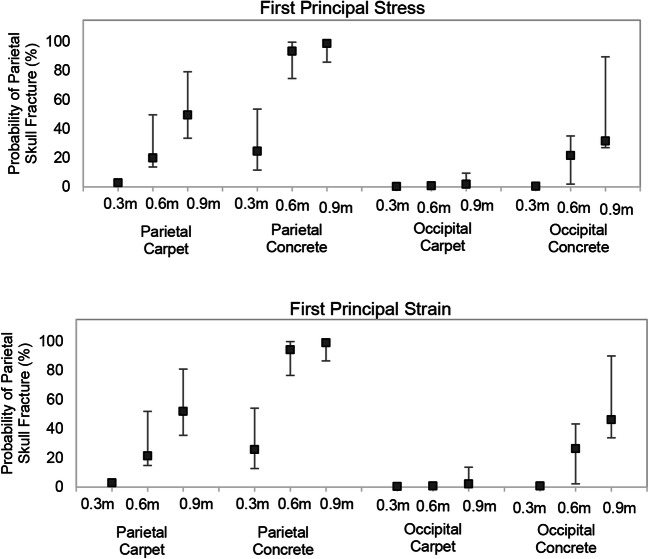
Median (squares) and minimum/maximum range (error bars) of probability of parietal skull fracture due to parietal and occipital head-first impacts in falls from 0.3, 0.6, and 0.9 m height onto concrete and carpet. Probabilities were obtained from the Q2 injury risk curves using first principal stress (top) and first principal strain (bottom)

The peak first principal stress and strain associated with the minimum, median, and maximum head impact force from low-height falls is given in Table S1. Using these values in conjunction with Q2 of the fracture risk curves (Fig. 5), we determined the range of probability of parietal skull fracture in different fall conditions (Fig. 6 and Table S2). We found that falls from 0.6 to 0.9 m onto the concrete contacting parietal bone had a high probability of skull fracture (75–100% and 86–100%, respectively) on the ipsilateral or impacted side. Falls from 0.9 m onto the carpet also had a moderate probability of skull fracture (34–81%). Falls from 0.3 m onto either the carpet or concrete had a low probability of parietal skull fracture (0–1% and 12–54%, respectively). Not surprisingly, the probability of parietal fracture on the contralateral or side opposite from the impact was very low (< 10%), regardless of fall height or impact surface. Interestingly, occipital impacts had the potential to cause skull fracture at the edge of parietal bones in falls from 0.9 m onto the concrete (34–90%). Because we had no cases with occipital fracture, we did not develop skull fracture thresholds for the occiput bone, and cannot predict the likelihood of occipital fracture under any of these circumstances.

## Discussion

Clinical differentiation of accidental and abusive head injury etiologies has proven to be a persistent problem for physicians. Given the paucity of documented pediatric accidental cases in the literature, the 11 well-witnessed, real-world infant falls in our clinical dataset, along with kinematics estimated from anthropomorphic surrogate event reconstructions and biomechanical parameters from FE simulations, provide the biomechanics community with valuable data that can facilitate validation of future computational models of infant head injuries. In addition, kinematics measured in our controlled surrogate drops and biomechanical parameters from corresponding FE simulations representing fall scenarios involving three heights, two surfaces, and two head impact locations, can serve as a data set for future studies investigating the likelihood of head injury in low-height falls.

To develop skull fracture risk curves, we used anthropomorphic surrogate studies and multiple biomechanical response parameters from FE simulations. Potential of using these biomechanical parameters to be used as skull fracture predictors in infants were assessed not only in terms of quality of fit of the corresponding developed risk curves and their prediction accuracy rates but also by comparing the distribution of these parameters with the actual location and pattern of skull fracture in real-world falls. An additional large set of controlled head-first fall drop tests [13] were used to evaluate the likelihood of parietal skull fracture in a variety of common low-height household fall settings using our developed risk curves and corresponding threshold values. The robustness of our fracture likelihood predictions in all controlled fall scenarios is evident in the consistency across predictions using our risk curves based upon first principal stress and strain. In addition, the similarity between Q1, Q2, and Q3 curves and corresponding threshold values for each of the skull fracture predictors suggests that uncertainties accompanied by real-world reconstruction may not affect the injury risk curves too much. In summary, we find that falls with parietal impact from 0.9 m have high potential to result in fracture regardless of the impact surface; however, those from 0.6 m are prone to more surface-dependent variability. Importantly, we also demonstrate possibility of parietal fracture following 0.9 m falls onto the concrete with occipital impact.

### Fracture risk thresholds: first principal stress and strain

Using surrogate and FE reconstructions of real-world fall accidents, we found skull fracture thresholds similar to failure stress and strain values reported for infant parietal skull [30]. Our 50 and 95% fracture risk thresholds for first principal stress (25.229 and 36.015 MPa, respectively) and for first principal strain (0.0464 and 0.0669, respectively) compare favorably to the average ultimate stress and strain values (30.95 and 0.072 MPa, respectively) measured by Coats and Margulies [30] in infant parietal bone from subjects 19 days–4.5 months of age.

**Fig. 7:**
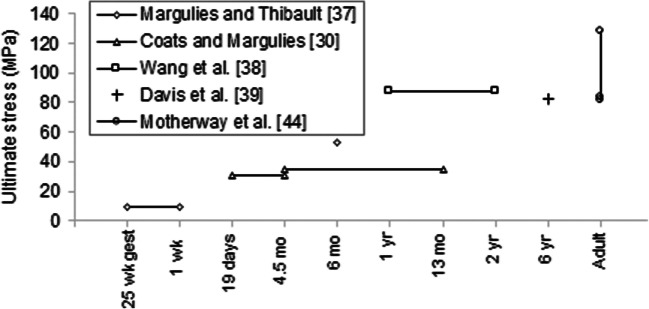
Ultimate stresses reported in the literature for human pediatric and adult parietal bone via three- and four-point bending

When placed in context with ultimate stresses reported via three- and four-point bending of pediatric crania of varying ages, our first principal stress fracture threshold (25.229–36.015 MPa) and Coats and Margulies’ ultimate stress (30.95 MPa) underscore the order of magnitude range in skull fracture thresholds for ultimate stress over the age spectrum from infant to adults, with ultimate stress increasing dramatically during the first year, then asymptoting to adulthood (Fig. 7). Margulies and Thibault [37] reported an ultimate stress averaging 9.3 MPa for 25 weeks of gestation to 1-week-old parietal samples and 52.8 MPa for 6-month-old parietal samples; Coats and Margulies [30] reported a parietal ultimate stress averaging 35.08 MPa for 4.5–13 months old; Wang et al. [38] reported 87.12 MPa for 1–2 year-old parietal samples; and Davis et al. [39] reported 82.87 MPa for 6-year-old frontal and parietal samples without distinguishing between the skull plates. Although Klinich et al. [40] estimated the 50% fracture risk thresholds for first principal stress to be 55 and 41 MPa for the inner and outer tables respectively in the 6-month-old cranium, their values were derived from a FE model of human dimensions constructed using porcine skull properties, rather than from direct measurements of the human skull. Therefore, these data were excluded from the comparison. In a similar manner, Li et al. [25] used “reverse engineering” to derive the 50% fracture risk thresholds for first principal stress to be 8.1 MPa, 10.7 MPa, 13.4 MPa, and 16.1 MPa for 0-, 3-, 6-, and 9-month-old infants, respectively. The elastic modulus used for the cranium in their 0–9 month-old FE head model was not determined via any measurements, but rather optimized to match peak head accelerations in Prange et al.’s cadaver drop tests [17]. Such optimization yielded a cranial elastic modulus (164.3 MPa) much lower than the actual elastic moduli measured in infant parietal (533.43 MPa) and occipital (407.27 MPa) skulls, restricting the subject age range from 19 days to 4.5 months old [30]. The low elastic modulus used in Li et al.’s model may explain their low fracture thresholds for first principal stress. These pediatric data were also excluded from Fig. 7.

Several studies have tested the adult skull in tension or compression. Evans and Lissner [41] reported the ultimate stress of parietal bone to be 70.53 MPa in tension and 152.2 MPa in compression without stating the strain rate; McElhaney et al. [42] reported the ultimate stress for cranial bone to be 43.44 MPa in tension and 96.53 MPa in compression at the constant velocity of 0.01 in/min; Wood [43] reported the ultimate tensile stress for cranial bone to be 48.26 and 127.6 MPa at strain rates 0.005 and 150 s^−1^, respectively. Of note, only Motherway et al. [44] performed three-point bending failure tests and found the ultimate stress of 62–97 year-old parietal samples to be 83.48 MPa, 81.04 MPa, and 128.1 MPa at the loading rates of 0.5 m/s, 1.0 m/s, and 2.5 m/s, respectively. The similarity between these values and ultimate stresses reported for 1–2 year-old and 6-year-old crania by Wang et al. [38] and Davis et al. [39], respectively, suggests that after rapid increases in ultimate stress between birth and 6 months, failure properties for the pediatric skull begin to approach those of the adult skull by age 6 years.

In both the pediatric and adult literature, ultimate strain measurements are surprisingly sparse. Wang et al. [38] reported an ultimate strain of 0.0866 for parietal samples from children 1–2 year olds, Davis et al. [39] reported 0.033 for frontal and parietal samples from a single 6-year-old cadaver, and McElhaney et al. [42] reported an ultimate compressive strain of 0.051 for cranial bone samples from adults 56–73 years old, without differentiating between skull plates. Comparing these reported strains to our first principal strain 95% fracture risk threshold (0.0669) for 0–6 month-old infants and Coats and Margulies’ [30] ultimate strain for 1.5-month-old infants (0.072), we conclude that ultimate strain of parietal bone in infants may not vary with age as much as ultimate stress. This conclusion agrees with Coats and Margulies [27] who found no significant effect of age on the ultimate strain of infant cranial bone.

In addition, in all simulated fall scenarios, we identify consistent predictions for parietal fracture likelihood and fracture pattern using first principal stress and strain as skull fracture metrics. Coats et al. [23] used first principal stress to validate their 1.5-month-old model’s ability to predict fracture as this was found to be the best predictor of failure from simulations of in vitro mechanical tests [24]. Roth et al. [19, 20] and Miyazaki et al. [5] used von Mises stress in the validation of fracture behavior in their 6-month-old, 17-day-old, and 23-month-old infant head models without justifying its advantage over other biomechanical parameters. Although Roth et al. [20] demonstrated good correspondence with acceleration traces reported in Prange et al.’s [17] 30-cm drops, they only used their 6-month-old and 17-day-old models to simulate a single fracture case each. As a result, they were unable to identify tolerance thresholds for skull fracture. Furthermore, Miyazaki et al. used a single parameter, von Mises stress, as a fracture threshold (17.6 MPa). Reported by Ommaya et al. [45], this value was purported to be the ultimate stress of a single “young child” sample with a 4-mm-thick skull. Cao et al. [21] and Zhou et al. [22] each examined two parameters in their 10-year-old models (Cao et al. used von Mises stress and strain; Zhou et al. used von Mises stress and first principal strain), but could not determine fracture thresholds because they only simulated a single case without fracture. Their maximal values were well below ultimate stresses and strains reported for 6-year-old and adult crania [39, 42, 43]. Li et al. [25] adjusted their model simulations until von Mises stress distributions matched fracture patterns in Weber’s cadaver tests, and not surprisingly, when they evaluated multiple metrics (first principal stress and strain, maximal shear stress, and von Mises stress), concluded that von Mises stress demonstrated the highest fracture prediction accuracy rate. In the current report, we adjusted head impact locations to optimize agreement between distributions of all four potential predictors and the fracture patterns in all seven of our clinical cases with fracture, to eliminate bias towards any single response parameter.

### Parietal fracture prediction for falls with parietal impact

To date, the present study represents the first FE model to assess likelihood of infant skull fracture in a variety of low-height fall scenarios with the aid of injury criteria developed based on real-world fall cases and validated against ultimate stress and strain values derived from mechanical tests on infant skull cadavers. Previously, we reported that peak head impact forces for 0.9 m falls onto carpet approach those for concrete, but peak head impact forces for 0.6 m falls onto carpet were significantly lower than those for concrete [13]. In the current report, we find that while falls from 0.9 m with parietal impact exhibit high probabilities of parietal fracture and falls from 0.3 m exhibit low probabilities of parietal fracture for both the carpet and concrete, falls from 0.6 m exhibit surface-dependent variation in skull fracture risk, with falls onto the concrete demonstrating higher probabilities than those onto the carpet. Importantly, our skull fracture risk curves are further validated against published infant cadaver studies, as they would predict the presence of parietal fracture in all 15 of Weber’s [15] infant cadaver (< 8.2 months old) drop tests from 0.82 m onto a stone tile, carpet, or linoleum. In addition, we predict a significantly lower risk of skull fracture below 0.3 m, consistent with no skull fracture reported by Prange et al. [17] in infant cadaver drops from 0.15 and 0.3 m onto a metal anvil.

Examining published clinical studies on pediatric head injuries following low-height falls, we focus on those limited to young children and infants. Among the children ≤ 2 years old who experienced non-inflicted head injuries in Duhaime et al.’s [8] prospective study, skull fractures were observed in 43 (57%) of the 76 subjects, and linear skull fractures were equally likely to occur from falls < 1.2 m and those > 1.2 m or falls downstairs. Pooling our findings in Fig. 6 across all fall heights and both impact surfaces for parietal impact, we would predict 51.2 ± 37.0% and 50.4 ± 37.5% risk of parietal skull fracture for falls ≤ 0.9 m using first principal stress and strain, respectively. Ibrahim et al. [9] reported a skull fracture frequency of 73% among 67 hospitalized infants (0–12 months old) who had experienced falls ≤ 0.9 m. We predict 75–100% probability from falls 0.6–0.9 m onto the concrete. Finally, in a retrospective study of 149 children ≤ 2 years old experiencing accidental head injury due to falls from 0.15 to 6 m, Thomas et al. [10] found a significant increase in the mean fall height in cases with skull fracture or intracranial injury (1.32 ± 0.28 m) than those without (1.01 ± 0.22 m). This parallels our predictions that the probability of skull fracture is significantly greater at 0.9 than 0.3 m.

### Parietal fracture prediction for falls with occipital impact

Both first principal stress and strain predictors in our study indicate probabilities of parietal fracture for 0.9 m falls onto concrete with occipital impact. The bone-suture junction is less structurally rigid than the skull plate in infants [30] that makes the infant skull more flexible and also allows the skull plates to move relative to each other. Therefore, the likelihood of parietal skull fracture due to occipital impact can be a result of bone flexure or relative movement of skull plates.

Studies published nearly 70 years ago reported fractures on the external skull surface in regions of outward bending and on the internal surface in regions of inward bending. While inward bending always occurred under the site of impact, outward bending sometimes occurred at considerable distances away from the site of impact [46, 47]. Given the thinner skull and lower elastic modulus [30], the infant skull has a lower structural rigidity than the adult skull and might be expected to more frequently experience fractures away from the impact site. Bilateral fractures have been observed in clinical case reports [36] and in both human and porcine infant cadaver drop tests. In several of Weber’s [15, 16] human infant cadaver drops with parietal-occipital impact, two parietal fracture lines were reported. While most of these were unilateral parietal fractures, the fracture lines in one case were biparietal and discontinuous at the sagittal suture. Similarly, in drop tests of porcine infant specimens (2–17 days old) onto a rigid aluminum surface with parietal impact, Powell et al. [48] observed no occipital fracture but observed parietal fractures that initiated at the coronal suture, away from the point of impact. Drop heights were not reported. Our present study is the first to propose fracture risk curves for pediatric skull fractures away from the site of impact.

### Limitations

Although this study significantly advances pediatric head trauma literature by proposing new biomechanical fracture criteria and predicting parietal fracture in low-height fall scenarios, the limitation of our study is the lack of any occipital fractures in our clinical data set. As a consequence, we were unable to develop injury criteria for occipital fracture or to predict occipital fracture in fall scenarios. However, making the assumption that principal stress and strain fracture thresholds are similar for parietal and occipital skull plates, we would predict a low probability of fracture for the three occipital impact cases in Table 1 (case 94: 2–6%, case 98: 11–38%, and case 108: 0–1%); in actuality, none of these cases had radiological evidence of fracture. Given the lower ultimate stress and strain of infant occipital bone compared to parietal bone but increased thickness of the occipital plate [30], future studies should collect additional cases with occipital skull fracture in falls in infants and investigate whether the occipital fracture threshold following parietal or occipital impact are higher than reported here for parietal fracture.

One additional important consideration with our parietal skull fracture risk curves is that a sideways fall often concludes with shoulder or limb contact before the head. Our whole-body surrogate reconstructions of short falls simulated worst-case risk scenarios with head-first contact. Thus, the likelihood of skull fracture risk might differ from those reported here if the fall were “broken” by contact of another body part during the fall.

## Conclusions

We developed parietal skull fracture risk curves for infants under 5.5 months old through reconstruction of real-world accidental falls in infants. To minimize the uncertainties accompanied by accident reconstruction procedure, rigorous inclusion criteria were employed during data collection process, impact kinematics were estimated through whole-body anthropomorphic surrogate reconstructions, and precise and comprehensive approaches were used to verify the reliability and precision of surrogate and FE reconstructions. FE simulations were validated against not only the peak impact forces but also the entire impact force-time histories measured in surrogate reconstruction drops. As an improvement over the existing accident reconstruction studies, FE simulations were also verified by considering stress and strain distribution in terms of the location and pattern of skull fracture. Among all four potential predictors, maximal first principal stress and strain best correlated with the occurrence of parietal skull fracture, and their corresponding proposed tolerances are in agreement with published in vitro material failure tests. Finally, the resulting risk curves were used to evaluate the worst-case likelihood of parietal skull fracture in head-first, low-height infant falls. We conclude that the likelihood of parietal skull fracture in head-first falls from 0.3 m is very low, regardless of fall condition. Falling from 0.9 m onto the concrete on either occipital or parietal site can result in parietal skull fracture. An improvement over anecdotal clinical reports and heuristic evidence, the outcomes of the present study can be used to more accurately assess likelihood of skull fracture in infant falls for a variety of fall heights, impact locations, and contact surfaces.

## Electronic supplementary material

ESM 1 (DOCX 15 kb)

ESM 2 (PNG 518 kb)

High resolution image (EPS 205668 kb)
