# Supplementary material for: Infant skull fracture risk for low height falls
Source: Int J Legal Med. 2018 Sep 7;133(3):847–62. doi: 10.1007/s00414-018-1918-1 (PMC6469693; doi:10.1007/s00414-018-1918-1)
Supplement: Supplementary file 1 — (DOCX 15 kb) [file 414_2018_1918_MOESM1_ESM.docx]

**Table S1.** Maximal values of each parietal skull fracture predictor due to parietal or occipital impact falls from 0.3, 0.6 and 0.9 m onto carpet and concrete. These values extracted from the FE simulations represent the median head impact force and the minimum and maximum (in parenthesis).

|  | | | **First principal**  **strain** | | **First principal stress (MPa)** | **Shear stress (MPa)** | **Von Mises stress (MPa)** | |  |
| --- | --- | --- | --- | --- | --- | --- | --- | --- | --- |
| **Parietal**  **Carpet** | $\left\{ \begin{aligned} \\ \begin{aligned} \\ \\ \begin{aligned} \\ \begin{aligned} \\ \\ \\ \end{aligned} \end{aligned} \end{aligned} \end{aligned} \right.$ | 0.3m | 0.022 (0.018-0.025) | 12.42 (10.11-13.67) | | 6.83 (5.99-7.36) | | 12.60 (10.96-13.68) | |
|  |  | 0.6m | 0.037 (0.034-0.057) | 20.16 (18.50-25.19) | | 10.08 (9.25-12.60) | | 19.44 (17.79-24.49) | |
|  |  | 0.9m | 0.047 (0.042-0.057) | 25.19 (22.73-30.18) | | 12.60 (11.37-15.09) | | 22.01 (24.49-29.62) | |
|  |  |  |  |  | |  | |  | |
| **Parietal**  **Concrete** | $\left\{ \begin{aligned} \\ \\ \begin{aligned} \\ \begin{aligned} \\ \\ \\ \end{aligned} \end{aligned} \end{aligned} \right.$ | 0.3m | 0.039 (0.033-0.048) | 21.14 (17.79-25.77) | | 10.57 (8.89-12.89) | | 20.45 (17.55-24.65) | |
|  |  | 0.6m | 0.066 (0.055-0.092) | 35.12 (29.21-48.27) | | 17.56(14.61-24.13) | | 34.59 (28.73-48.16) | |
|  |  | 0.9m | 0.079 (0.059-0.096) | 41.86 (31.89-50.57) | | 20.93 (15.94-25.28) | | 41.16 (30.94-50.21) | |
|  |  |  |  |  | |  | |  | |
| **Occipital**  **Carpet** | $\left\{ \begin{aligned} \\ \begin{aligned} \\ \\ \\ \\ \\ \end{aligned} \end{aligned} \right.$ | 0.3m | 0.009 (0.007-0.011) | 5.23 (3.98-6.08) | | 3.45 (2.64-3.72) | | 6.36 (4.66-7.28) | |
|  |  | 0.6m | 0.014 (0.012-0.015) | 7.45 (6.42-8.36) | | 5.13 (3.91-6.23) | | 9.56 (7.77-11.57) | |
|  |  | 0.9m | 0.020 (0.016-0.034) | 10.81 (9.04-16.96) | | 8.94 (6.89-12.61) | | 16.85 (12.84-24.53) | |
|  |  |  |  |  | |  | |  | |
| **Occipital Concrete** | $\left\{ \begin{aligned} \\ \begin{aligned} \\ \\ \\ \\ \\ \end{aligned} \end{aligned} \right.$ | 0.3m | 0.013 (0.010-0.018) | 6.80 (5.60-9.53) | | 6.36 (4.38-8.20) | | 11.40 (8.38-14.86) | |
|  |  | 0.6m | 0.039 (0.020-0.045) | 20.55 (10.83-22.98) | | 18.45 (9.94-17.41) | | 35.01 (18.17-32.62) | |
|  |  | 0.9m | 0.045 (0.042-0.062) | 22.43 (21.60-33.17) | | 19.53 (18.68-20.75) | | 37.45 (35.62-40.78) | |

**Table S2.** Probabilities of parietal skull fracture in parietal or occipital impact falls from 0.3, 0.6 and 0.9 m onto carpet and concrete. These probabilities were determined from FE simulations using the median head impact force, and the minimum and maximum head impact forces (in parenthesis).

| **Fall condition** | **Skull fracture probabilities based on maximal principal**  **Stress (%)** | | | **Skull fracture probabilities based**  **on maximal principal**  **strain** | | |
| --- | --- | --- | --- | --- | --- | --- |
| **Fall height**  **Impact location & surface** | 0.3m | 0.6m | 0.9m | 0.3m | 0.6m | 0.9m |
| **Parietal Carpet** | 3  (2-4) | 20  (14-50) | 50  (34-79) | 3  (2-5) | 21  (15-52) | 52  (36-81) |
| **Parietal Concrete** | 25  (12-54) | 94  (75-100) | 99  (86-100) | 26  (13-54) | 94  (77-100) | 99  (87-100) |
| **Occipital Carpet** | 0  (0-1) | 1  (1-1) | 2  (1-9) | 0  (0-1) | 1  (1-1) | 2  (1-14) |
| **Occipital Concrete** | 0  (0-1) | 22  (2-35) | 32  (27-90) | 1  (1-2) | 26  (2-43) | 46  (34-90) |
