# Supplementary figures and images for: Infant skull fracture risk for low height falls
Source: Int J Legal Med. 2018 Sep 7;133(3):847–62. doi: 10.1007/s00414-018-1918-1 (PMC6469693; doi:10.1007/s00414-018-1918-1)

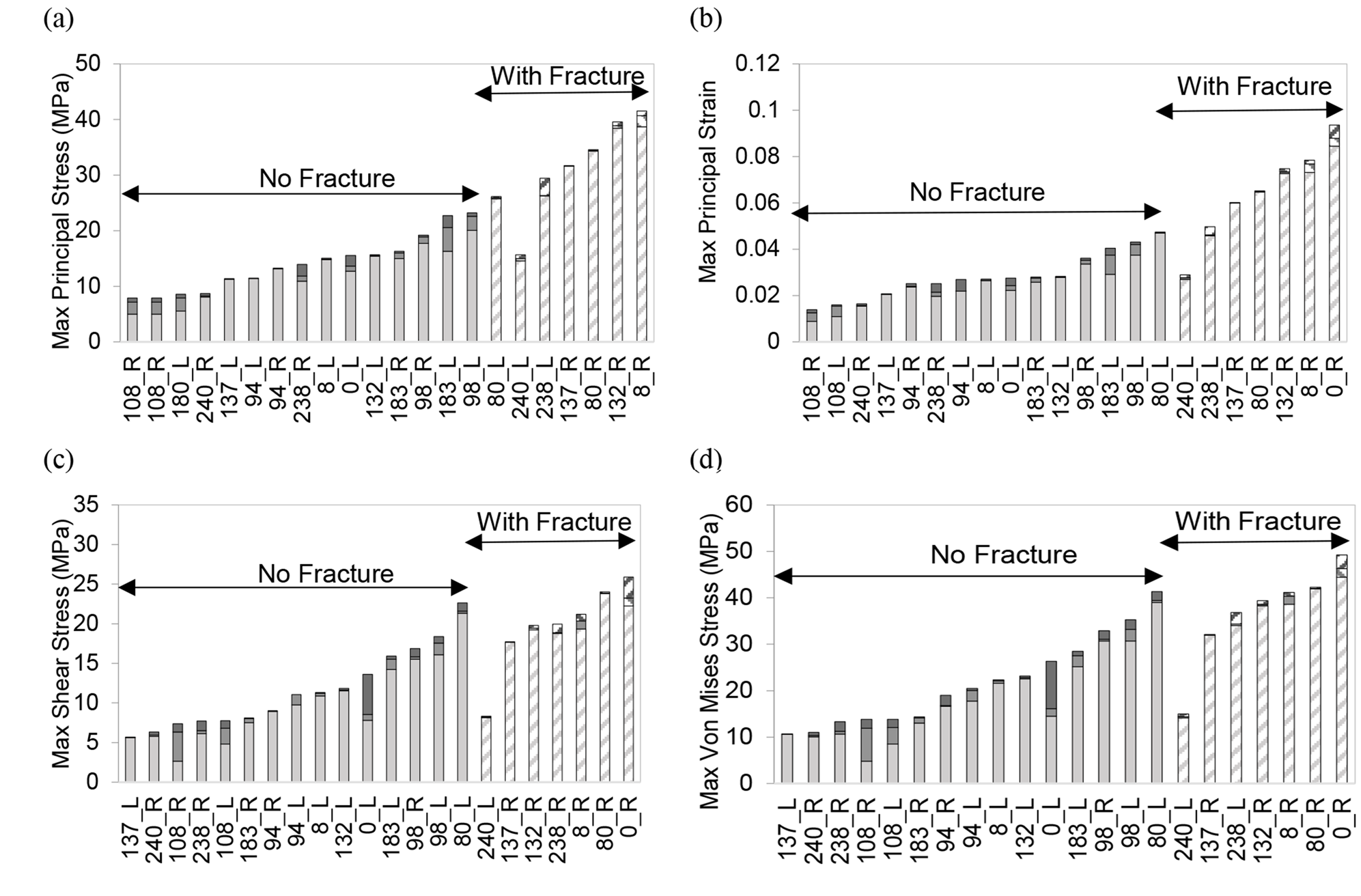

Supplement: Supplementary file 2 — (PNG 518 kb) [file 414_2018_1918_Fig8_HTML.png]
